# Comment on “Some Aspects of Nonbeverage Alcohol Consumption in the Former Soviet Union”

**DOI:** 10.1155/2015/604219

**Published:** 2015-07-26

**Authors:** Y. E. Razvodovsky

**Affiliations:** Grodno State Medical University, 230009 Grodno, Belarus

The paper by Jargin [1] addressed highly relevant topic: the phenomenon of nonbeverage or surrogate alcohol consumption. In Russia, the consumption of homemade spirits (samogon) and surrogate alcohols (i.e., products that contain alcohol but are not intended for consumption), also referred to as noncommercial or unrecorded alcohol, is somewhat common [2, 3]. Among the most common surrogates are industrial spirits, antiseptics, lighter fluid, and medications containing alcohol. Unrecorded alcohol also includes alcohol produced by distilleries but sold without the payment of taxes and counterfeit beverages that are passed off as commercial brands. According to WHO statistics [4], unrecorded alcohol accounts for one-third of all consumption in Russia, while some estimates show a figure as high as 50% [5]. One study indicated that the prevalence of past year surrogate alcohol consumption in an average Russian city was 7.3% [2]. A recent study found that the vast majority of Russians cited their lower cost and greater availability as important reasons for drinking noncommercial alcohol beverages [6]. This is why the low-income groups of the population and heaviest drinkers in Russia are the likeliest to consume noncommercial alcohol, and this effect is intensified during periods of economic recession [7].

It also seems very likely that illegally distilled, counterfeit, and surrogate alcohol poses a risk to human health, playing an important role in the high level of alcohol-related deaths in Russia. It was found that home-made spirits contained the toxic alcohols that could cause damage to the liver [2]. The findings from Izhevsk (Russia) study indicated that among working-age males who reported surrogate use, the relative risk of dying from causes directly related to problem drinking (e.g., alcoholic psychosis, alcoholic cardiomyopathy, alcoholic liver cirrhosis, and acute alcohol poisoning) was 25.5 in relation to those who consumed only legal alcoholic beverages [8].

Historically, government policies designed to raise prices and restrict availability of commercial alcohol beverages in Russia have driven black market growth. Surrogates consumption increased markedly following the prohibition of vodka sales in July 1914 as Russia mobilized for war [9]. A similar rapid rise in consumption of illicitly produced alcohol and surrogates has occurred during Gorbachev's antialcohol campaign in the mid-1980s [10].

The development of events on the Russian alcohol scene in recent years shows how complex and multifaceted the problem of undocumented alcohol consumption is. In 2006, amendments were introduced to the Federal Law “On State Regulation of the Production of Ethyl Alcohol, Alcohol and Alcohol-Containing Products” number 171, which seriously impacted the alcohol situation in the country [11]. Firstly, the share capital for manufacturers and retailers of alcohol products was substantially increased, as a result of which many small manufacturers and traders were pushed out of the alcohol market. Secondly, the Unified State Automated Information System (USAIS) for electronic registration of alcohol was introduced. Thirdly, there was a change in excise stamps. The lack of coordination of the activities of various agencies during the implementation of these innovations led to a temporary shortage of legal vodka. This especially impacted the Russian hinterland since, there, the supply of alcohol to the population was mainly carried out by private entrepreneurs who were forced to stop their operations [11]. Under the conditions of excessive demand, the lack of alcohol provoked an epidemic of poisonings by surrogates (industrial fluid, antiseptics, and household chemicals). Thousands of people (primarily heavy drinkers) in various regions of Russia ended up in hospitals with the diagnosis of “toxic hepatitis” [12].

Making vodka less affordable through differential taxation was an essential element of the Russian alcohol policy in the most recent years [13]. The government's new excise policies have made legal alcohol in the country more expensive. Over the past several years, Russia's alcohol prices have grown nearly three times [13]. An official alcohol sales figure has been steady on the descent for the past few years as prices soared. According to Rosstat, total alcohol sales decreased by 18,9 percent from 2005 to 2013, while vodka sales fell by 35,1 percent. The negative outcome of these measures was that the rising price of vodka generated a demand for unrecorded alcohol. Over the last years, the shift to noncommercial alcohol has accelerated because many Russians lack the funds to pay for the legal alcohol as consumer purchasing power has decreased [6].

According to experts' estimates, the market of surrogate alcohol over the past six years has been up from 500 million liters to 800 million liters [13]. Many official retail outlets, in particular, those in remote provinces, tend to sell bootleg vodka. In many rural areas, which do not have stores selling alcohol, there are informal networks for the sale of unrecorded alcohol from hand to hand [13]. This development points out to the danger of using alcohol tax policy in the alcohol market, which is not fully controlled by the government.

The major problem is that the informal alcohol market is largely immune to regulation and effective policymaking. The Russian government should consider a number of potentially effective approaches to addressing the problem of noncommercial alcohol, including raising public awareness of the risks of surrogates drinking and creating an alternative to strong alcoholic drinks by preferences to low alcoholic beverages.
